# Design Requirements for a Digital Aid to Support Adults With Mild Learning Disabilities During Clinical Consultations: Qualitative Study With Experts

**DOI:** 10.2196/10449

**Published:** 2019-03-04

**Authors:** Ryan Colin Gibson, Matt-Mouley Bouamrane, Mark Dunlop

**Affiliations:** 1 Digital Health and Well-Being Group Department of Computer & Information Sciences University of Strathclyde Glasgow United Kingdom

**Keywords:** learning disabilities, intellectual disability, communicative disorder, augmentative and alternative communications systems, primary care

## Abstract

**Background:**

Adults with mild learning disabilities (MLDs) face a plethora of obstacles when accessing effective health care. Central to many of these barriers is communication, with medical practitioners often remaining untrained on how to interact with patients who have learning disabilities (LDs). To date, research on how to promote this communication has largely centered on the development of low-tech aids.

**Objective:**

The objective of this study was to assess the feasibility of utilizing tablet technologies to promote communication between general practitioners and patients with MLDs. We achieved this by identifying a set of design requirements from experts in LDs.

**Methods:**

A set of design guidelines was formed during a 2-phase process. Phase 1 involved conducting a series of requirements-gathering interviews with 10 experts in LDs—the protocol of which emerged from the results of a separate scoping review. The interviews were subjected to a framework analysis to discern the key requirements discussed by the experts, and these were embedded within a technology probe. In phase 2, this probe was presented to a subset (n=4) of the experts during a round of usability studies, and the feedback received was used to update the requirements identified in phase 1.

**Results:**

An initial set of design requirements has been produced that may assist in the development of clinical Alternative and Augmentative Communication technologies for adults with MLDs. Factors that must be considered range from the health, physical and cognitive needs of stakeholders, to the more individual needs of users.

**Conclusions:**

The experts involved in the study were optimistic about the proposed app. They believe that such technologies can help to alleviate time constraints and promote communication by presenting information in a form understood by both practitioners and patients.

## Introduction

### Background

Since the turn of the millennium, international policies [1] have been introduced that compel mainstream services to offer access to improved and unprejudiced care. Consequently, an increase in the well-being of those affected by learning disabilities (LDs) has been recognized [2]; however, their life expectancy remains far below that of the general population [3]. This suggests that the quality of care being administered remains suboptimal, with previous literature identifying a variety of barriers that patients with LDs face when accessing health care services [4,5]. One of the most widely cited barriers affecting this standard of care is the breakdown in communication between medical professionals and patients.

Howells suggests that the “art of general practice lies in the ability to communicate with patients” [6,7]. However, people with LDs have a variety of impairments that influence their ability to participate in conversations [8]. First of all, cognitive impairments affect an individual’s ability to learn, meaning patients are likely to have a restricted knowledge of the human body and may be unable to recognize the presence of certain medical conditions [9]. Their expressive skills may also be affected, and this impedes their ability to comprehensibly describe the symptoms that they do acknowledge. On the other hand, people with LDs often have better receptive skills [8] and will have more success acquiring the information being conveyed by a general practitioner (GP), provided complex concepts such as medical jargon are avoided—an issue that is prominent throughout the clinical domain [10]. Impairments in abstract thinking and long-term memory [11] may hinder the patient’s ability to provide an accurate medical history, with GPs relying on caregivers to provide this information. However, patients often object to this process [11], and there is evidence to suggest that it leads to inaccurate information being extracted [8].

Patients with mild learning disabilities (MLDs) may utilize Alternative and Augmentative Communication (AAC) devices [12] to assist them in conveying their needs. To explore the prevalence of these technologies within the clinical domain, the authors have conducted a separate scoping review. The finer details of the study have been described previously [13]; however, the results indicate that despite the call for digital support being made by practitioners as far back as 1997 [14], low-tech solutions continue to be the primary means used to supplement communication. This contrasts significantly with other vulnerable populations [15,16] where Information and Communication Technology is used copiously to advance health literacy.

### Objectives

Moreover, 1 possible reason for this may be the lack of support available during the development of such technologies. We address this gap by investigating the potential use of tablet devices to promote communication between practitioners and patients with MLDs. Specifically, we have examined whether extracting information in advance of the consultation can have a positive impact on such communication. To achieve this, we used the results of the scoping review to shape 9 requirements-gathering interviews involving a purposive selection of experts in LDs. A technology probe was developed using this data and, subsequently, presented to a subset of the experts to further inform the extracted requirements. These requirements may be used to support researchers in the future development of medical AAC apps that cater to the complex needs of adults with MLDs. In addition, the findings made may also help to support the general population in communicating medical information to practitioners, as vulnerable patients are often considered as a litmus test to the effectiveness of interventions [17]. Throughout, we intend to answer the following research questions (RQs):

RQ1: What do adults with MLDs and GPs require from an aid that aims to support them during clinical consultations?

RQ2: What impact may mobile devices have on the clinical consultation process?

RQ3: What are the design guidelines for medical AAC apps that assist adults with MLDs?

## Methods

This study employed a 2-phase design process. The first phase focused on the development of a technology probe using the requirements extracted from experts in LDs during a round of semistructured interviews. In phase 2, the probe was evaluated by a subset of these experts to further inform the requirements identified. Both phases were conducted under ethical approval from the Department of Computer and Information Sciences Ethics Committee at the University of Strathclyde (ID CIS470, CIS614). We will first present an overview of the project before describing the design process used in more depth.

### Project Overview: Medical Research Council Complex Interventions Framework

The research presented in this paper is part of a wider project to develop, in conjunction with the views of stakeholders, a tablet app to promote communication between GPs and patients with MLDs. In this context, the term “mild learning disability” may be applied to an individual if they satisfy the following criteria as listed by the World Health Organization [18]: “they have a significantly reduced ability to understand new or complex information and to learn and apply new skills. This results in a reduced ability to cope independently and begins before adulthood with a lasting effect on development.” Those with MLDs are generally able to communicate their needs but may struggle with complex ideas such as medical symptoms.

**Figure 1:**
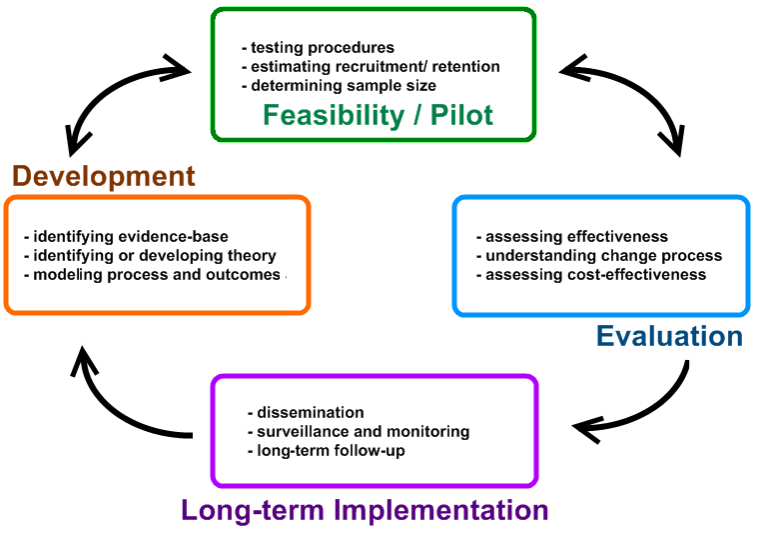
Medical Research Council framework for complex interventions.

To ensure the proposed aid is developed in a systematic manner, the authors are following the Medical Research Council’s Framework for Complex Interventions [19], as shown in Figure 1.

Our decision to utilize the Complex Intervention Framework may be justified via the following 3 criteria:

1. As discussed previously, people with MLDs tend to have impaired higher order cognitive skills [11] and may find it less challenging to discuss their requirements when interacting with artifacts as opposed to developing them from scratch. The iterative nature of the framework supports this process by offering multiple opportunities to present a probe to stakeholders for evaluation and subsequently update its design based on the results achieved.
2. Great emphasis is placed on the collection of evidence. This is important as it ensures that the researchers assess whether the developed product caters to the wide range of needs and impairments present in adults who have MLDs.
3. The framework is widely approved throughout the clinical domain, meaning that a product developed using these steps is more likely to be accepted within current practice.

The first stage (“Development”) has almost come to its conclusion. We have established an evidence base for the proposed app via the aforementioned scoping review [13]. This review highlighted that low-tech AAC devices continue to be the primary form of support provided to patients with MLDs, despite the call for the implementation of high-tech devices being made as far back as two decades ago. Furthermore, AAC technologies are yet to be embedded within common practice, meaning even low-tech devices differ in terms of their availability and functionality across health boards and individual practices. As such, there is a clear need to develop a resource that can be adopted on a national scale.

The next substep is to establish how the intervention may fit into and improve current practice. This has been partially achieved via the research presented in this paper because it describes the development and evaluation of a technology probe based on the requirements identified by 10 experts in LDs. During future research, adults with MLDs will be given the opportunity to interact with and subsequently shape the design of the probe in preparation for stage 2. The “Feasibility and Piloting” and “Evaluation” stages will require the intervention to be embedded within the clinical domain and its performance monitored over a short period of time. This will allow the researchers to determine whether the app is having its desired effect and subsequently make improvements before a long-term evaluation study is carried out in stage 4.

### Phase 1: Requirements Gathering

#### Design and Setting

Phase 1 involved identifying an initial set of requirements for a tablet app that supports adults with MLDs in communicating symptoms to their GP. To achieve this, the lead author conducted semistructured interviews with 10 experts in LDs. At the time, RCG was a master’s student and had 1-year experience in conducting qualitative research. The protocol used was modeled around the themes that emerged from the aforementioned scoping review [13], and this will be discussed in the Procedure subsection. All interviews were carried out at locations convenient to the participants, and no monetary rewards were provided because they occurred during working hours.

Our decision to include experts, as opposed to adults with MLDs, centered on the following reasons:

1. People with LDs often have impaired higher order cognitive skills such as abstraction [11] and may find it difficult to envisage how the proposed app can assist them in conveying information to their GP.
2. Stakeholders are often unaware of their needs during the early design stages of a product, and their true requirements do not become known until they have interacted with a concrete artifact [20].

As such, it was appropriate to involve experts first as they were able to identify various accessibility issues that may be mitigated before a concrete probe is presented to the people with MLDs. We plan to include participants with MLDs in future research and will update the guidelines presented in this paper accordingly. This process should lead to representative requirements being extracted from patients with MLDs.

#### Participants

The target sample size was set between 10 and 15 participants to account for data saturation [21] and to ensure a wide range of knowledge and expertise was utilized throughout the design stage. The recruitment process involved the first (RCG) and second authors (MMB) contacting various LD charities, academics, and government agencies via telephone and email throughout the city of Glasgow. A total of 10 participants consented to take part (6 females and 4 males), at which point recruitment ceased as we had reached our target sample size. All participants were interviewed separately apart from participants 1 and 2 (see Table 1), as it was convenient for them to be interviewed together.

#### Procedure

Before commencing, participants had all questions resolved by RCG, and written consent was obtained. The interviews were then conducted on a semistructured basis to allow stakeholders the opportunity to raise and expand upon topics outside of the protocol. RCG presented 6 sets of questions based on the themes that emerged during the scoping review [13], including potential communication barriers, the communication modalities utilized by people with LDs, the communication aids encountered by the experts, potential barriers to AAC technologies, professionals’ attitudes toward people with LDs, and personalization. Additional questions relating to the aesthetics and features of the proposed app were also presented.

In addition, GPs were required to discuss their overall experience and confidence in consulting with patients with LDs. The question sets presented to the participants are provided in Multimedia Appendix 1. On completion of the interviews, the experts were asked to raise any topics that had not been addressed throughout. The sessions were recorded with participant consent, and the mean duration was approximately 34 min—ranging from 25 min to 1 hour.

#### Data Analysis

The lead author transcribed the recorded interviews to further their understanding of the captured data. The transcriptions were then subjected to a framework analysis [22,23] to produce a structured summary of the requirements discussed by the experts. First, an initial thematic framework was developed by RCG based on the themes and subthemes that emerged throughout the scoping review. On further inspection of the transcribed data, the lead author recognized that some of the concepts discussed did not conform to these topics, because of the semistructured nature of the interviews. Further codes were therefore created to address this information. RCG then grouped similar codes together to form overarching themes, at which point MMB (who has extensive experience conducting qualitative research) reviewed the developed framework, and any discrepancies were resolved by MDD.

**Table 1:** The demographics of the participants interviewed.

ID	Profession	Sex
1	Governmental advisor—gathers evidence for the Scottish Government on the health inequalities experienced by those who have LDs^a^; previous support worker for people with LDs	Female
2	Governmental advisor involved in the coproduction of policies affecting those who have LDs; previous support worker	Female
3	Full-time support worker for an LD charity	Female
4	Academic in social work and social policy	Female
5	Governmental advisor involved in promoting Scotland’s “Keys to life” strategy	Male
6	General practitioner	Male
7	General practitioner	Male
8	Academic in inclusive education; previous deputy head teacher for a special needs school	Male
9	Academic in cognitive psychology; developed accessible information resources for the National Health Service	Female
10	Academic in aging, frailty, and dementia; previously involved with a national LD charity	Female

^a^LD: learning disability.

**Table 2:** The symptoms to be selected by the participants during the usability studies.

ID	Symptoms
1	The participant is suffering from toothache caused by tooth decay.
2	The participant is not in pain. Instead, they hear ringing sounds and feel dizzy and sick. They are experiencing tinnitus.

The resulting framework was utilized by RCG to code the transcriptions, and the tagged excerpts were transferred to their appropriate positions in the framework analysis table. This table has been made available in [13].

### Phase 2: Usability Study

#### Design and Setting

In preparation for phase 2, the lead author used the design requirements identified in the previous phase to develop a technology probe of the proposed app. A technology probe may be considered as a representation of a device that is utilized by stakeholders to inspire the design process through exposure to new experiences [24]. These stakeholders are, therefore, able to shape the design of the final artifact by interacting with the probe and commenting on their experiences.

To ensure adults with MLDs can interact with the probe during future research, a subset of the experts described in Table 1 were required to participate in a usability study. The experts completed 2 tasks using the probe and commented on the features they felt were accessible to the LD population and those that may present barriers. This enables the researchers to mitigate potential accessibility barriers before the introduction of stakeholders who have mild LDs. Once again, the study was conducted by RCG at a location convenient to the participant, and no monetary rewards were provided.

#### Participants

On the basis of the guidelines for iterative design by Dumas and Redice [25], the sample size was set between 3 and 5 participants. This supports the researchers in addressing key design and flaws over a short period of time, rather than having to carry out an extensive number of studies to obtain similar information. Invitations to participate were sent out to the experts involved in phase 1, as they had prior knowledge of the project and understood what the probes goals were. Participants 1, 2, 4, and 8 in Table 1 consented to take part, at which point recruitment ceased as the target number of participants had been met.

#### Procedure

The participants were required to work through the questionnaire embedded within the probe and select symptoms relating to 2 distinct medical conditions. These conditions (shown in Table 2) were designed to ensure that the experts explored all features within the app. Furthermore, no assistance was provided during this process, except when the experts explicitly asked for help or were unable to advance within the app. This ensured that the lead author refrained from influencing the actions of participants and that key design flaws were naturally identified [25]. Any points of indecision were also observed and noted by RCG to be explored further at the end of the session.

Once the experts had finished selecting the symptoms for both conditions, they were prompted to give their views on the probe, and it is appropriateness for the MLD population. The feedback received was then used to refine the requirements extracted during the previous phase. Over 1 hour of audio data were captured with participant consent, with each session averaging 21 min. A copy of the questions presented and an explanation of the conditions chosen are provided in Multimedia Appendix 2.

#### Data Analysis

To extract the features deemed to be accessible to the LD population, as well as those that may be improved on, the transcriptions were subjected to the same framework analysis process described in phase 1. A copy of the framework analysis table may be found in [13].

## Results

### Requirements

Throughout the semistructured interviews, a number of requirements were discussed by the experts, which helped to shape the design of a technology probe for the proposed app. In this section, the key requirements will be introduced and are supported by the excerpts contained within the resulting framework analysis table found in [13]. The rows in the table are organized to reflect the participant IDs found in Table 2, with the exception that the views of participants 1 and 2 have been combined into 1 row (2) because they were interviewed together.

### Communication Challenges

#### Barriers to Communication

Both of the GPs interviewed cited communication difficulties as the primary barrier to effective care for patients with MLDs. They suggested that 2 factors play a prominent role in this breakdown in communication, the first of which involves the patient’s interpretation of a condition. People with LDs are often undereducated on both the human body [9] and their own health needs and may, therefore, misinterpret or fail to recognize the presence of symptoms. The second factor centers on the inability (of all stakeholders) to describe conditions in a clear manner [26], as discussed by participant 7:

The [patient’s] understanding of their condition, their interpretation of symptoms, [and] their ability to communicate symptoms may be different. Our ability on the practitioner’s side to elicit those symptoms may be different or more challenging. Ultimately a consultation is based around two-way communication and at times aspects of that communication can be difficult. Whether it be to do with comprehension or to do with abstract thinking or just basic communication.

#### Implementing Accessible Language

Potential strategies discussed by the experts to improve this communication focused largely on the language used by stakeholders. First, 4 of the participants stressed the need to utilize clear and simplistic language and avoid medical jargon where possible. Strydom et al came to a similar conclusion while evaluating the accessibility of medical information leaflets; however, they established that some complex terms (such as brand names) were crucial to patient’s comprehension [27]. This suggests that developers of medical AAC apps should consider the views of potential users when creating this information to ensure it is understood as intended.

Moreover, 3 further participants revealed that people with LDs often find it difficult to answer broad, open-ended questions such as “How have you been feeling?” Instead, the questions presented should be closed and focus on solitary ideas to first break the consultation down into manageable chunks and then ease the cognitive load placed on patients.

#### Utilizing a Range of Modalities

People with LDs are at an increased risk of being unable to understand the language used to describe concepts; thus, technologies must use alternative formats to represent this information [27]. The experts cited several communication modalities that, when combined, may be effective in achieving this, and these will be described in the next subsection.

### Communication Modalities

Adults with MLDs are heterogeneous in nature and may not respond to information in the same manner as others [28] - for example, 40% have hearing impairments [29] and can find it difficult to understand data transmitted via sound. To overcome this issue, the experts suggested targeting a variety of communication modalities to ensure an individual’s complex needs are catered to.

#### Pictures

The bulk of the experts suggested that imagery is the most effective modality used to convey information (and therefore promote discussion) providing it immediately captures the concept being depicted. Furthermore, 2 primary reasons that were suggested for this included being easier to process than words alone [30] and being available throughout the entire process. In a variety of health-related studies, patient comprehension has been proven to increase when resources conveyed information using both imagery and text [27,31,32]. In addition, participant 9 revealed that pictures can act as a referent and assist in overcoming potential short-term memory impairments:

[By] having a kind of visual record in front of somebody [it helps to] keep track of where they are. Concrete things are very helpful if there’s something there that can be pointed to as a reminder or help to keep a focus.

#### Speech

A multitude of requirements will have to be met by the images embedded within the app to be effective for all users. As such, this information will have to be conveyed in an alternative format to cater to those users who do not understand the meaning behind a particular image. Of the useful modalities described by the experts, 1 was speech, providing the individual needs and abilities of adults with MLDs are taken into consideration. Participant 3 revealed that the communication skills of this population can vary widely but suggested that the use of accessible language guidelines can help to mitigate this issue.

The experts discussed 2 ways in which speech may be incorporated into the digital aid: (1) accepting speech as user input to forgo the reliance on touch screens and (2) playing back the text displayed on the screen. To ensure this process is accessible, the volume, style, and pace in which the speech is returned should be made customizable.

#### Accommodating for a Range of Users

Combining speech, text, and imagery to represent medical conditions should increase patient comprehension as they may use the modality that makes sense to them when presented with each potential option. This can lead to an increase in the accuracy of the data being collected and may also be beneficial to the general population, with many patients concluding that the language used by practitioners is both inappropriate and confusing [33].

### Simplistic Interface

#### Limiting Clicks

Operational difficulties [34-36] have resulted in AAC abandonment rates rising to as high as 53.3% [35], with users preferring to revert to traditional forms of communication as opposed to persisting with complex technologies. The experts, therefore, stressed the need to develop simplistic user interfaces and suggested that a reduction in both the complexity and number of steps involved in a process could assist in achieving this, as discussed by participant 10:

It would depend on how easy the [tablet application] was to use but the quicker the better I would say. The shorter the better in terms of how much time someone would have to [complete it]. So, easy to use absolutely, [with] as few steps in the process - as few clicks in the process as possible.

The experts highlighted 1 method to reduce the number of steps involved in the app, which involved mitigating the number of irrelevant questions being presented. Consequently, a dynamic-based questionnaire should be implemented, with questions being adapted to suit the specific health needs of the patient. This closely mimics the consultation process described by participant 7:

I think the first question would be hi how can I help you today? How are you getting on? How are you managing? And then each subsequent question depends on that.

#### Limiting Choice

All experts agreed that the amount of choice available to adults with MLDs should be reduced to ease the cognitive load placed on users. Nevertheless, they had conflicting views on the maximum number of options present at any 1 time. Participant 9 suggested that this population is often excluded from the decision-making process and are more inclined to answer yes or no questions. As such, the number of options available should be reduced to a minimum and built upon a consistent framework:

So maybe keeping [the] options limited and building [the questionnaire] out in a kind of structure so that when you get to the end point you might have to go the long route rather than the shortcut.

In contrast, several of the other experts felt that this population could cope with greater choice, with up to 4 potential options being cited. Furthermore, participant 8 discussed the need to prioritize adaptable technologies that alter the number of options displayed on screen:

Some people might cope with quite a large volume of information and some might need very little - you know two or three items...My recommendation would be that [the app] was very flexible [and] could adapt to the individual needs of a person.

### Individualization

In this study, 7 of the experts stated that AAC technologies should be able to adapt to the characteristics of the user, as summed up by participant 1:

I think just to highlight one of the things that was said is that it’s not a one size fits all approach, you should tailor it to each individual’s needs.

Some of the requirements described previously strive to achieve this. For example, conveying information via speech, text, and imagery will enable patients to use the modality best suited to their needs. In addition, implementing an adaptive questionnaire will ensure that the questions being presented are suited to the patient’s individual health needs. Finally, modifications to the tablet device itself can help cater to more individual needs, such as updating the screen sensitivity settings to account for motor impairments [37].

#### Adapting the Look of the App

Further opportunities for customization centered on the ability to change the aesthetics of the aid, which includes adapting the number of options displayed on screen, as discussed by participant 9. In addition, 4 of the experts revealed that many adults with LDs have an impaired perception of color and may require specific color schemes to assist in the comprehension of text, as summed up by participant 4:

Yellow is the kind of standard [background color]. But normally if someone needs a different color for whatever reason they’ll tell you. So, I don’t know if that’s something that you [can] change [in the app].

#### Overcustomization

Although there are great benefits to adapting technologies to cater to the individual needs of users, participant 8 emphasized the dangers of overcustomization:

I do worry about things getting too individualized, you know, so that it can’t be shared in any way.

Developers should, therefore, consider the ability to share such technologies across a range of stakeholders and refrain from simply tailoring the app to address the needs of 1 user group. Vanderheiden et al [38] have explored this issue in the past and have concluded that the characteristics and needs of potential subgroups of users can be readily identified. As such, they advocate for interfaces that adapt to the type of user operating the system to mitigate the accessibility issues common to that population. This could potentially entail saving the accessibility preferences of an individual and reloading them during future interactions with the device.

### Questions

#### Target-Specific Health Demographics

The health demographics of adults with MLDs differ dramatically from that of the general population [29,39]. Consequently, this evidence must be used to justify the symptoms that are embedded within the aid to ensure the questions presented are relevant to the user’s condition, as discussed by participant 2:

The content needs to be informed by the specific health experiences of people with learning disabilities. People with learning disabilities have different patterns of diseases to people in the general population…different kinds of cancers for example are more prevalent.

GPs often overshadow many of the common conditions experienced by people with LDs, for example, hearing impairments [39,40]. The app, therefore, has the potential to draw greater attention to these conditions and increase their rate of diagnosis.

#### Question Types

The GPs interviewed also discussed a range of information they deemed essential to the formulation of a diagnosis. Participant 6 briefly described the first 5 questions they would explore during a consultation:

The first thing I’d ask is why are they here today? Then whatever they describe you ask for duration, if that has happened before and if there are any other symptoms. And [then finally] how they are in general.

This led to the development of 4 question sets that should be explored by medical AAC technologies:

1. Questions to extract the symptoms experienced by the patient
2. Questions to determine the duration and intensity of symptoms
3. Questions to extract the history of symptoms
4. Questions that extract the overall health of patients, particularly focusing on their mental well-being as the National Institute for Health and Care Excellence estimates that 40% of adults with LDs have undiagnosed mental health problems [41]

### Patient Histories

Besides effective communication, the success of consultations involving adults with MLDs may rely heavily on the availability and accuracy of patient histories, as described by participant 6:

...the second thing you tend to utilize is previous records. For example, if they have [had] a particular health problem then you can anticipate certain problems [occurring]. History from their carer or family members often gives you cues to work beyond.

From this excerpt, you may assume that all symptoms selected throughout the aid should be stored for subsequent retrieval. However, participant 7 believes that this is not necessary and instead only the most significant symptoms should be stored:

Our role is largely an interpretive role translating people’s symptoms, alongside any investigations [and] what we know about the probability of a conditions prevalence etc. into a formulation of what’s going on. So to that extent I don’t always document every single symptom and I don’t know how helpful that might be.

The GP must, therefore, have access to the most significant symptoms selected by the patient when using the app.

### Requirements Gathering Summary

Further requirements are presented in Multimedia Appendix 3 and a summary of those discussed in depth are presented in Table 3. The participant ID of the experts who raised each requirement is also included to highlight the frequency in which they were proposed.

### Technology Probe Design

The Complex Intervention Framework states that a product must first be piloted before a long-term evaluation is carried out within its target environment. In preparation for this pilot study, a technology probe was developed using the requirements listed in Table 3 and subsequently evaluated by 4 of the experts listed in Table 1. This allows us to mitigate potential accessibility issues before the probe is introduced to stakeholders who have mild LDs. The decisions made during the development of the probe will be now be discussed; however, it is important to note that its functionality focuses solely on the features utilized by patients, meaning that features used exclusively by practitioners have not been implemented. This section is presented in 2 parts: (1) a description of the techniques used to adapt the probe to the individual needs of users and (2) a discussion on the development of a specialized user interface.

### Adaptability

#### Portability

From the offset, portability was prioritized as 1 of the most important features of the app. Consequently, we developed the probe using HTML5, CSS3, PHP, and JavaScript to be cross-platform. As a result, 1 version of the code may run on any device, and this has a considerable advantage over native apps as stakeholders are not restricted by the type of tablet in use. As such, they may utilize the device best suited to their needs, for example, those who have significant visual impairments may require a larger tablet to allow for objects to be increased in size. Medical practices may also purchase the tablet they deem to be most appropriate, thus increasing their likelihood to invest in the intervention.

#### Stack-Based Questionnaire

The need to limit the number of irrelevant questions being presented to patients with MLDs was also discussed in depth by the experts. To achieve this, an adaptive stack-based questionnaire has been implemented similar to that proposed by Bouamrane et al [42]. A main questionnaire stack is created based on the primary symptom selected by the patient—for example, pain in their eye. This stack contains the questions deemed vital to extracting the current health status of the patient, which means all the questions are presented to the user. The questions are removed one at a time from the top of the stack and presented, in order, provided the user upholds certain preconditions. The answers provided by the patients may then result in additional questions being added to the top of the stack. For example, the questions that have been designed to extract the symptoms of blepharitis may only be presented if the patient indicates that they have itchy, red eyes. Consequently, the adaptive questionnaire can reduce significantly the number of irrelevant questions being presented, as many are only added to the stack once the user has selected a specific symptom.

### User Interface

To present the questions contained in the stack to the patient, a specialized user interface was developed using the requirements listed in Table 3. This subsection presents a brief overview of the key design decisions made while developing this interface.

#### Trimodal Options

**Figure 2:**
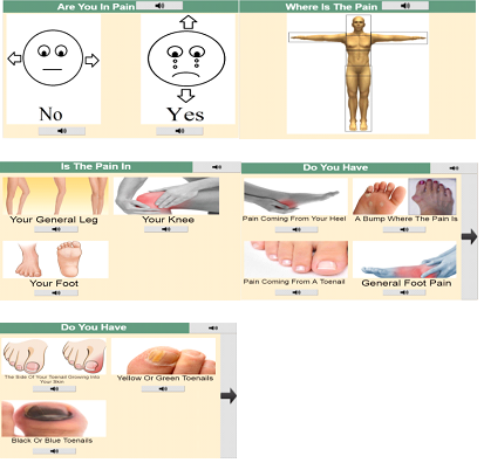
Specialized interface developed using the requirements listed by experts.

As shown in Figure 2, all options available to stakeholders have been conveyed via the use of 3 communication modalities. This includes pictures that closely match the options available, simplified text that provides a description of the symptoms presented, and audio that may be accessed in 2 manners. The user may request the program to sequentially highlight and playback all passages of text displayed on completion of page loads or simply select a particular audio button to have an individual passage played back. Patients may then utilize the modality that makes sense to them when presented with an option, thus increasing user comprehension. However, it is important to note that the images embedded within this probe are considered as placeholders. We intend to develop a set of resources in conjunction with the views of target stakeholders (during future studies) to ensure their complex needs are met [43].

**Table 3:** A summary of the requirements identified during the semistructured interviews.

ID	Requirement description	Participant ID
1	Text used to convey symptoms should be developed in conjunction with the views of target stakeholders. Medical jargon should primarily be avoided but some phrases (such as brand names) may be crucial to user comprehension.	2, 3, 8, 10
2	A variety of communication modalities should be targeted. As a result, symptoms should be represented by text, speech, and images where appropriate.	1, 3-5, 7-10
3	Images should be immediately identifiable to the user and subsequently developed in conjunction with the views of target stakeholders.	5, 8
4	The user should have the option to have text played back to them. The pace, style, and volume in which the text is played back should be customizable to suit an individual’s needs.	2-5, 8
5	The design of the app should be consistent throughout. An example may be embedding a help button at the top left-hand corner of all pages.	4, 9, 10
6	Questions presented to the user should be concise, straightforward, and focus on solitary ideas. All potential options should focus on a single subject.	1, 2, 4
7	The number of clicks used throughout the aid should be reduced to a minimum to aid users who have limited attention spans, etc.	10
8	A dynamic questionnaire should be implemented. Future questions should be shaped by the information previously supplied by the user.	7, 9
9	The number of potential options displayed on screen should be limited to a maximum of 4.	3, 4, 9, 10
10	The aid should port easily across various operating systems and screen sizes.	8, 10
11	The aesthetics of the aid should be made customizable to address the complex needs of stakeholders. The content should remain unchanged.	4, 5, 8, 10
12	The symptoms presented to stakeholders should be informed by the specific health needs of adults with learning disabilities, rather than that of the general population.	1, 2, 10
13	Questions should aim to extract the symptoms experienced by patients, the duration and history of these symptoms, and the overall health of patients.	6, 7
14	Questions should be presented one at a time.	3, 4, 9, 10
15	A minimum font size of 14 should be used throughout. Text should be made as large as possible.	3-5, 8, 9
16	Contrasting colors should be used to ensure information stands out and can be processed easily. The user should be able to select the color scheme that addresses their needs best.	3-5, 8, 10
17	The aid should provide symptoms experienced by patients in advance of consultations.	2, 4, 5, 7
18	Significant symptoms identified by the app should be stored for future retrieval by general practitioners. This will require the personal details of patients to be captured to act as keys within a database.	6-7
19	All feedback provided should be simple and constructive with a consistent help feature available to increase autonomy.	9
20	The overall consultation process should be broken down into manageable chunks.	1,2, 4

#### Simplifying the Consultation Process

To be effective, the experts suggested that the app should target those conditions commonly experienced by people with LDs. However, this could result in an overly complex questionnaire containing an abundance of questions, as there is evidence to suggest that this population is susceptible to a wide range of medical conditions [29,39,40]. The adaptive questionnaire described previously assists in reducing the number of questions presented as only those relevant to the patient’s condition are considered. 2 further strategies are used to reduce the cognitive load being placed on the user. The first image in Figure 2 contains a page that determines whether the patient is in pain. This enables a host of conditions to be disregarded immediately as many are placed exclusively into a pain or nonpain category.

In addition, different combinations of symptoms may be used to deduce the presence of a condition. Presenting all possible symptoms on screen at once could be cognitively challenging for people with LDs due to the amount of choice available to them. As such, the app restricts the maximum number of options displayed to 4, as shown in the fourth image of Figure 2. As a by-product, this strategy caters to those stakeholders who have significant motor or visual impairments as the area of space allocated to text/clickable objects may be increased. All questions presented also focus on solitary ideas to allow patients to focus on the particular areas of their health that are a cause of concern for them.

#### Designating an Area of Concern

As discussed in the previous section, adults with MLDs respond particularly well to concrete objects that they may point to. Hence, when a patient is required to indicate the body part causing them distress, an image of the body is presented. Nevertheless, this process relies heavily on the user possessing the motor abilities required to tap on small sections of the screen, for example, when selecting the left foot. Due to the prominence of motor impairments in those who have LDs, the probe prompts the user to confirm their selection by presenting all body parts situated in the proximity of the tap (shown in image 3 of Figure 2). This also enables those that were unavailable for selection in the original image, for example, the back, to be presented.

#### Skipping Questions

Forcing patients into selecting 1 of the options displayed may result in practitioners using incorrect information to form a diagnosis. Consequently, a skip button (shown in the right-hand side of image 4 in Figure 2) has been developed with the needs of the majority of stakeholders taking into consideration. As text may not be relied upon to convey information [27], the button makes use of an arrow to represent naturally the ability to move onto the next question/page. The success of this image will be discussed in depth in the next section. Once the questionnaire has been completed, a summary page will be presented for use by the GP. A more detailed description of the interface may be found in the study by Gibson et al [44].

### Technology Probe Evaluation Results

To update the extracted requirements, a series of usability tests were carried out on the probe by a subset of the experts described in Table 1. Participants 1, 2, 4, and 8 partook in the study, and the resulting framework analysis table has been made available in [13]. Row 2 reflects the views of expert 8, row 3 experts 1 and 2 (as they were interviewed together), and row 4 expert 4. Throughout this section, we will discuss the features deemed to be appropriate for people with MLDs, as well as those that may be improved upon.

#### Focus

One of the primary barriers expressed by the experts was the overall complexity of the consultation process. To gauge the patient’s health needs, GPs often use general open-ended questions such as “How may I help you?”; however, people with LDs tend to find it difficult to answer this style of question. Participant 4 believes that the probe can mitigate this issue by presenting short, closed questions that allow the patient to focus on a particular aspect of their health:

If you give someone [with LDs] a blank canvas to start off with their mind just goes blank and they don’t know where to begin. I think this is a good way to focus people for the conversation...I just think it would really help someone to clarify what points they want to convey.

Participant 4 also suggested that the app could help patients to rehearse the information they wish to convey, thus increasing their confidence to address the practitioner:

The carer [and the individual] could sit and go over this together and it could actually give them more confidence when they went in [to the appointment] ‘cause I think sometimes people feel quite intimidated. Some GPs don’t have the best bedside manner, so it gives someone the confidence to actually get their points across.

#### Consultation Times

There is evidence to suggest that consultations involving patients with LDs are heavily restricted by time [45], and this may affect the standard of care being provided. A total of 3 experts felt that the aid could alleviate time constraints by allowing the GPs to shape their questions based on the information collected outside of the appointment, as described by participant 4:

I think a lot of GPs now have extended consultation times for people with learning disabilities but that would mean they could make the most of that time rather than spending the first half of it trying to figure out what the person’s symptoms were.

#### Accessible Summary Page

Participant 4 discussed the need to include a second summary page in a format that is accessible to people with LDs:

It would be quite a respectful [and] empowering thing for the patient to have a summary of [the symptoms to] use when they go in for the consultation. So, the GP gets the summary, but the person also has a little prompt for themselves in terms of all the things they were feeling.

**Figure 3:**
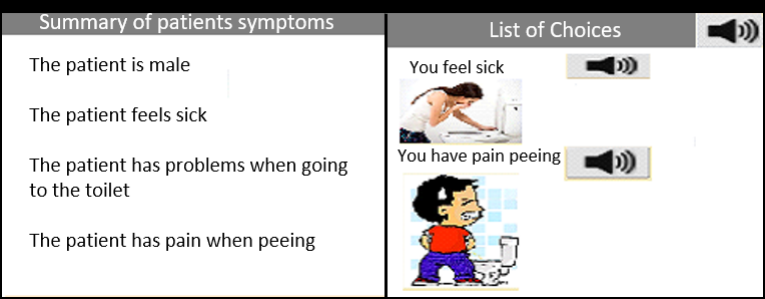
Summary pages for general practitioners and patients.

One way to achieve this is shown in Figure 3, where the options are represented by the 3 modalities discussed previously.

#### Communication Modalities

The placeholders used throughout the probe were deemed on the whole to be appropriate for adults with MLDs. All of the experts agreed that the combination of pictures, text, and speech is crucial to the patient’s understanding of the symptoms displayed. However, some aspects may be improved upon. Expert 8 believed that some patients could have difficulty understanding the more abstract symptoms, such as tinnitus:

...the one about tinnitus, for example, “do your ears feel stuffed up” they might not know how to describe it.”

This quote emphasizes the need to develop the resources used to convey symptoms in conjunction with target stakeholders to ensure they are understood as intended.

#### Conveying a Range of Conditions

A total of 2 experts were concerned about the meaning conveyed by various images and felt that some could be taken literally by patients with MLDs, as highlighted by expert 1:

...the skin one though...people might be very literal in their interpretation i.e. [my condition] doesn’t look like that, [so] it’s wrong to click that.

**Figure 4:**
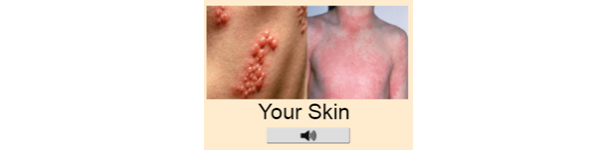
Image originally used to depict skin conditions.

Patients who have other skin conditions, such as eczema, may refrain from selecting the image shown in Figure 4 as their condition looks different to those displayed. Therefore, a more appropriate alternative would be to display a general image of skin to encourage individuals with any skin condition to select the option.

#### Highlighting the Skip Buttons Purpose

One feature within the app was deemed inappropriate for people with LDs. The skip button (shown in Figure 2) was developed with the use of an arrow to ensure all stakeholders, including those who have difficulty reading, could profit from its use. However, all 4 experts failed to select the button when required to do so, citing that its purpose was unclear. This led to the first author intervening and explaining that the button is used to skip the current question and subsequently present further options, at which point, its intention became clear, as discussed by participant 4:

See when you point it out it’s like of course it’s obvious but I suppose I didn’t automatically register that arrow was there. I do think that someone with a learning disability might find that tricky. So, you look at the options and then you have to make a connection between none of them and knowing that you have to press that button to get more options.

Much of the advice on how to improve the skip button, therefore, focused on making its purpose clear. Participant 4 suggested that a help feature should be implemented across all pages to ensure patients are able to obtain advice when unsure about how to progress, and this matches previous accessibility guidelines such as those provided by Medhi et al [46]. Once again, the information should be presented in an appropriate format with previous literature proposing the use of avatars and videos to deliver such content [15,47]. Further suggestions on potential improvements are presented in Multimedia Appendix 3.

#### Individualization

A total of 3 participants revealed that the opportunity to change the color schemes used is crucial to addressing the more individual needs of patients, as highlighted by participant 4:

That might be a good idea [changing the background color] because, depending on what the persons particular issue/condition is, there are certain colors that work better.

A range of impairments may also be catered to by altering the pace, style, and volume in which speech is returned. However, it is important to note that the content within the questionnaire should remain the same to all users, and this will be presented in greater depth within the Discussion section.

### Additional Features

#### Return Function

All participants disclosed the need to supply a return function to ensure any mistakes made by the patients can be rectified. Experts 1 and 2 suggested that a confirm function could be embedded that enables patients to corroborate their choice, as discussed by expert 2:

I was wondering [if you could include] a box that says, “did you mean your sight, is that correct yes or no” and if no it would go back.

However, participant 4 felt that this strategy could become irritating for those users who are consistently selecting the correct option and instead advocated for a traditional return button that displays the previous page.

### Guidelines for Medical Alternative and Augmentative Communication Apps

Overall, the experts discussed 5 main improvements to the developed probe: (1) the implementation of an accessible summary page for patients, (2) utilizing general pictures to represent a range of permutations, (3) providing audio feedback for all functional units, (4) allowing the user to return to a previous page, and (5) using a pain scale to distinguish between pain and discomfort. These requirements have been combined with the most significant of those found in Table 3 to form a set of guidelines (Table 4) for the development of medical AAC apps that target the needs of adult patients with MLDs.

**Table 4:** Developed guidelines for the implementation of medical Alternative and Augmentative Communication apps that target adults with mild learning disabilities.

ID	Guideline description
1	The overall consultation process should be broken down into manageable chunks by presenting small, closed questions that focus on solitary ideas.
2	Questions should focus on the health needs of target stakeholders rather than that of the general population as these may differ greatly.
3	Questions should aim to extract the symptoms experienced by patients, the duration and history of these symptoms, and the overall health of patients.
4	Information provided by stakeholders should be used to shape future questions in an attempt to limit the number of irrelevant questions being presented.
5	Information should be conveyed via a range of communication modalities including simplified text, immediately identifiable imagery, and speech.
6	The language and imagery used to convey information should be developed in conjunction with target stakeholders to ensure they are understood as intended. In general, medical jargon should be avoided but this may not be the case for all situations, for example, the use of brand names.
7	General pictures should be used to represent options that have a range of permutations. For example, a picture of eyes may be used to represent visual deficiencies.
8	Appropriate pain scales (such as the Wong Baker Smiley Face Pain Scale) should be used to distinguish if the patient is experiencing discomfort or is in pain.
9	The number of options available to the user should be limited. We recommend a maximum of 4.
10	Elements should be large in size and spaced far apart to accommodate for potential visual and motor deficiencies.
11	Key navigational and decision points should not be conveyed solely with the use of text.
12	A consistent layout should always be provided including the option to access a help feature. The user should be able to navigate across the interface, in both directions via skip and return buttons.
13	The aesthetics of such aids should be customizable; however, the content should remain the same.
14	A record should be kept of all the key activities made within the aid. Both patients and medical staff should have access to this information, represented in a format suitable to them.
15	The software should be portable to ensure stakeholders use the device most suited to their needs.

## Discussion

### Current Use of Communication Aids in Medical Domains

An extensive amount of research has been carried out to identify the barriers to effective health care experienced by patients with MLDs [2,4,39,48]. This literature highlights the important role communication has throughout primary care, yet surprisingly, little scrutiny has been placed on the impact digital technologies may have in advancing the health literacy of this population. Related studies have instead focused on specific aspects of the care process, for example, gaining consent [49], administering medication [50], and preparing for a stay in hospital [51] or have focused on other medical fields/populations, for example, dentistry [52] and children with LDs [31]. Nevertheless, this cohort of research has produced some similar findings to our own, thus enhancing the impact of the guidelines proposed in Table 4.

### Utilizing the Most Appropriate Communication Strategy

The experts interviewed throughout this research (particularly the GPs) have highlighted that a breakdown in communication can occur when information is presented in an inappropriate manner. Both Furberg et al [49] and Menzies et al [52] came to a similar conclusion and suggested that this process can have a detrimental impact on the patient’s ability to give consent as the individual may not fully comprehend the options available or why a specific action is required. As with our app, these studies have therefore focused on simplifying the information to be presented and customizing the delivery of content to suit the individual requirements of the user.

In addition to implementing speech, identifiable imagery, and accessible language, Menzies et al [52] found that animation and video can be effective in conveying how procedures are carried out, including the tools used within them. This concept could also be used to capture those conditions that involve movement, such as pain when raising your arm, to ensure they are identifiable to patients with LDs. Furberg et al [49] also investigated the most effective style of imagery to embed within their decision support tool and found that over 40% of participants preferred cartoon graphics. The remaining participants were split between simplistic images and those that followed a graphic novel design, and this emphasizes that a range of needs must be considered when developing technologies for stakeholders who have LDs.

In addition to presenting data in an accessible manner, the dentists involved in [52] requested that such aids extract the patient’s likes, dislikes, and previous dental history in a manner similar to that of patient passports [53]. This strategy may promote communication significantly as the medical professional will be able to use the techniques most suited to the patient’s needs and has been explored in depth by Prior et al [54]. One final novel way of enhancing the capacity of a patient with LD to converse with a medical professional was explored by Hall et al [51]. They used virtual reality to embed the patient within a clinical environment, and this process resulted in participants retaining health-related information weeks after their exposure to the technology.

### Customization

Many of the modalities discussed in the previous subsection were also targeted by Salgado et al when identifying features for a mobile app that supports users in the management of medication [50]. Nevertheless, these authors explored the concept of customization in further depth. Interestingly, they recognized the need to change the interface based on the category of user interacting with the app. This property could be extremely useful for the proposed app as different and more complex information may be presented to the medical professional or caregiver supervising the patient. With regard to personalizing features to suit the needs of an individual, Salgado et al [50] agreed with our experts by suggesting that this process should be balanced with the development of features that promote independence and comprehension for a wide range of users.

Traditional AAC technologies often afford the user the ability to customize the number of options displayed on screen [55,56]. In contrast, several of the experts interviewed suggested that this population is often unaware of their information needs, and the customization process may be too complex for people with LDs. As such, they proposed that the maximum number of options displayed should be capped at 4 to ease the cognitive load placed on the individual. Further benefits of this include catering to visual and motor impairments as elements may be increased in size because of the screen space available and reducing the need for technology-specific actions such as scrolling. However, 1 downfall is the need to present additional questions to ensure the range of potential symptoms is displayed. In addition, the questionnaire should be based on the evidence available on the health demographics of people with LDs. Consequently, enabling the user to change the number of options displayed may result in the path to certain conditions being altered, meaning erroneous information could be captured.

Furthermore, the resources used to convey symptoms should be developed in conjunction with stakeholders to ensure their complex needs are catered to. As such, it does not make sense to allow users to edit these at will, and instead, a range of resources should be developed and made interchangeable to suit certain subgroups of users. Moreover, 2 further opportunities for customization include adapting the color schemes employed as well as the style, pace, and volume in which speech is returned. We plan to develop the features discussed with the use of participatory design techniques to ensure they are effective in achieving their goal. Stakeholders may then customize the interface to suit their own individual needs and impairments.

The aesthetics of the aid is certainly an important factor; however, it is not the sole driving force behind its success. The experts revealed that the questions presented to the user should be based on their own health needs. Consequently, a static questionnaire would be inappropriate as the patient would be required to answer an abundance of irrelevant questions when providing information about their condition—a process that may be particularly detrimental to those who have limited attention spans. Instead, a dynamic questionnaire was developed that adapts to the needs of the user, and this will be discussed in the next subsection.

### Presenting Appropriate Questions

The work presented in this paper is somewhat similar to that of the research carried out by Bostrom and Eriksson [31]. Consequently, many of the requirements identified across both studies were similar including simplistic screens that employ minimal information, the need to present 1 question at a time, limiting the number of interactions required to operate the aid, supplementing textual information with speech and images, implementing accessibility guidelines, and avoiding technology-specific actions such as swiping. Further requirements identified by these authors include offering breaks when the user is required to complete a lengthy process and supporting navigation via buttons that utilize left and right arrows [31].

The primary difference between the 2 studies is the length of the developed questionnaires. Bostrom and Eriksson included 43 questions within their aid, yet the experts interviewed by us suggested that such a length could be problematic for people with LDs because of a variety of reasons including cognitive impairments and short attention spans. Prior et al attempted to solve this obstacle in a project that aimed to extract the needs of adults with LDs during their admission to hospital [54]. They restricted the questions presented based on the user’s personal information such as their gender. We have built upon this concept by utilizing the symptoms extracted from the patient to shape future questions, and this was achieved via a dynamic stack-based questionnaire similar to that proposed by Bouamrane et al [42]. This process significantly reduces the number of irrelevant questions being presented as many are only asked provided a certain option has been chosen. It can also assist professionals in meeting current and future guidelines such as those presented in Sullivan et al [57]. Any new conditions found in these documents may be added to the stack via a subquestionnaire and subsequently brought to the attention of the GP when appropriate.

### Feasibility of Using Mobile Devices

By discussing the requirements listed by both previous literature and the experts interviewed, we have answered 2 of the research questions proposed. The final question centers on the feasibility of embedding mobile devices within consultations involving patients with LDs. This question may be split into 2 parts: how GPs will react to the use of mobile devices, and how accessible are mobile technologies to adults who have mild LDs.

The GPs involved in the study disclosed that they had never used mobile devices to obtain information; however, they were open to doing so provided it benefited the patient. Their main concern during this process was the accuracy of computer algorithms in discerning the current health status of an individual, yet this apprehension may be mitigated provided these algorithms are developed using robust methods. They also advocated for receiving information in advance of the consultation although they suggested that a diagnosis should not be provided as the final decision should be made by medical professionals.

In addition, 2 main barriers to the use of tablet technologies were discussed by the experts: the presence of motor/visual impairments and digital exclusion. These impairments may hinder the user’s ability to carry out touch screen–specific actions such as swiping, as well as their ability to tap on objects with the required accuracy. Rocha et al discussed these barriers in depth when exploring the accessibility of an iPad mini [58]. They found that the participants were able to learn how to operate the device relatively quickly; however, they struggled to grasp the concept of less intuitive operations. Furthermore, they experienced difficulties when performing actions that required fine motor skills, but their motivation to complete the tasks presented did not detract. Rocha et al also measured the error rate and time taken to complete 2 tasks on the tablet device in comparison to a traditional desktop setup [58]. They found that people with LDs were able to complete the tasks at a significantly faster rate and with greater accuracy while using the tablet. This bodes well for the potential use of such devices within clinical consultations.

### Limitations and Future Work

The authors made a deliberate decision to interview experts, as opposed to adults with MLDs, and the rationale behind this has been justified in the Methods section. As such, we argue that this is not a limitation of the study. However, we recognize that the number of GPs involved was restricted and that data saturation for this population has not been achieved. Although GPs may not be considered as experts in LDs, as many are undertrained on the needs of this population, it is important to consider their requirements during the development of the app. As a result, there is scope to interview further GPs until data saturation has occurred. Further opportunities for future work include creating an ontology to represent the conditions common to people with LDs and conducting codesign workshops with adults who have LDs to update the guidelines presented in this paper. Finally, a concrete representation of the aid should be embedded within the medical environment to determine the impact it may have, for example, in reducing consultation times and increasing the diagnosis of certain conditions.

### Conclusions

Our study has demonstrated the potential use of tablet technologies to promote discussion between practitioners and adults with MLDs. We developed the first representation of a high-tech research-based aid to achieve this by utilizing the extensive knowledge held by a variety of experts in LDs. This has resulted in the creation of a set of guidelines that will be instrumental in assisting developers in the future implementation of medical apps that cater to the complex needs of adults with MLDs.

It is important to consider a number of factors during the development of such technologies. First, the conditions embedded should exploit the evidence available on the health needs of people with LDs as their demographics differ significantly from that of the general population. Several modalities (including text, speech, and imagery) should be targeted to represent this information and should be developed in conjunction with the views of target stakeholders to increase user comprehension. Both the questions and options presented to patients should be limited to ease the cognitive load placed on adults with MLDs.

It is also important to develop features that cater to the wide range of physical and cognitive impairments that may be present in people with LDs. This process should be restricted to the customization of the aesthetics of the app and should refrain from extending to the content embedded within. Symptoms should be extracted in advance of the consultation to assist in mitigating time constraints, and the app should be portable to ensure patients are able to use the device best suited to their complex needs.

## Appendix

Multimedia Appendix 1 Question sets presented to participants during semistructured interviews.

Multimedia Appendix 2 Description of the conditions selected by the experts during the usability study and the questions proposed throughout.

Multimedia Appendix 3 Less relevant results discussed by experts.

## Abbreviations

AAC: Alternative and Augmentative Communication
GP: general practitioner
LD: learning disability
MLD: mild learning disability
RQ: research question
