# Supplementary material for: Design Requirements for a Digital Aid to Support Adults With Mild Learning Disabilities During Clinical Consultations: Qualitative Study With Experts
Source: JMIR Rehabil Assist Technol. 2019 Mar 4;6(1):e10449. doi: 10.2196/10449 (PMC6421513; doi:10.2196/10449)
Supplement: Multimedia Appendix 1 [file rehab_v6i1e10449_app1.pdf]

Question sets discussed with learning disability experts.

1. General background questions on what experience they have in working with people with learning disabilities, and what projects they're currently working on that benefit those who have learning disabilities.
2. Questions based on the stigmas shown towards people with learning disabilities.
3. Questions based on the awareness of the health needs of people with learning disabilities.
4. Questions based on the differences between having a conversation with someone who has a learning disability and someone who has not as well as the challenges involved.
5. Questions relating to the communication modalities used by people with learning disabilities.
6. Questions relating to the communication aids used by people with learning disabilities.
7. Questions relating to touch screen technologies and how proficient people with learning disabilities are in using them.
8. Questions relating to the specific size of tablet that should be used for the application.
9. Questions relating to the amount of answers that should be present per question asked i.e. how many would exceed the cognitive load possessed by people with learning disabilities.
10. Questions relating to the overall look and feel of the application i.e. colour schemes etc.
11. Questions based on the person's expectations of an application that assists people with learning disabilities during the consultation process.
12. General question to allow further issues to be brought up by the participant.

Question sets discussed with General Practitioners

1. General background questions on what experience they have in working with people with learning disabilities i.e. how often they consult with those who have learning disabilities.
2. Questions relating to the GPs confidence in consulting with those who have learning disabilities
3. Questions relating to the education that they have received on learning disabilities
4. Questions relating to the differences that occur when consulting with those who have learning disabilities compared to that of the general population
5. Questions relating to the communication aids used during consultations
6. Questions relating to any technologies used when consulting with the general population.
7. Questions relating to the communication modalities used by people with learning disabilities
8. Questions relating to where the application would be best used i.e. waiting room, during the consultation etc.
9. Questions relating to the possibility of storing the results produced by the application.

10. Questions relating to the types of information the application should collect from the user
11. Questions relating to the overall look and feel of the application.
12. General question to allow further issues to be brought up by the participant.
