# Supplementary material for: Design Requirements for a Digital Aid to Support Adults With Mild Learning Disabilities During Clinical Consultations: Qualitative Study With Experts
Source: JMIR Rehabil Assist Technol. 2019 Mar 4;6(1):e10449. doi: 10.2196/10449 (PMC6421513; doi:10.2196/10449)
Supplement: Multimedia Appendix 2 [file rehab_v6i1e10449_app2.pdf]

Questions presented to the experts at the end of the usability studies.

1. Do you think the app is appropriate for people with learning disabilities?
2. What features do you think help stakeholders to convey the symptoms they are experiencing?
3. Are there any features that may cause problems for the stakeholders?
4. Are the images and language used throughout appropriate?
5. Do you think the application is visually appealing to those who have learning disabilities?
6. How would you improve the application?
7. Is there anything else you would like to add?

Explanation of scenarios.

Scenario one aims to give the participant a brief introduction to the application. Interviewees were required to make use of the audio features embedded throughout and explore a condition relating to pain. This ensured that the experts utilised the body feature and are aware that the application requires the user to triangulate decisions that may be affected by motor deficiencies. All symptoms to be selected were immediately presented on screen meaning the skip function was not required within this scenario.

Scenario two aimed to explore those features that were not covered during the first. The participants were required to select symptoms relating to a condition in which pain is not prevalent. This enabled both sections of the application (pain and non-pain) to be scrutinised and for the subsequent language and pictures used to represent various manifestations to be reviewed by the experts. Not all options were immediately available to the participant, thus requiring them to identify and subsequently use the skip button in order to present further symptoms at key stages within the application.
