# Supplementary material for: Design Requirements for a Digital Aid to Support Adults With Mild Learning Disabilities During Clinical Consultations: Qualitative Study With Experts
Source: JMIR Rehabil Assist Technol. 2019 Mar 4;6(1):e10449. doi: 10.2196/10449 (PMC6421513; doi:10.2196/10449)
Supplement: Multimedia Appendix 3 [file rehab_v6i1e10449_app3.pdf]

Multimedia Appendix 3

This document contains those results that were deemed to be less important to the development of the application.

Results: Requirements

This section refers to the requirements listed by the 10 experts involved in the requirements gathering interviews and are referred to under the original headings embedded in the main manuscript.

Communication Challenges

Implementing Accessible Language

The language used to describe symptoms will not be suitable for all potential users, as discussed by participant 10; *"So if we say to someone "can you tell me where you are in pain?" the term "in pain" might not be the right term to use. And sometimes that's down to local colloquialisms."* As such, researchers should prioritize the development of technologies that are able to adapt to the needs of a range of users.

Communication Modalities

Pictures

Participant 4 suggested that the overuse of pictures could have a detrimental effect on the ability of stakeholders to use the application, *"I think there's a process isn't there in trying to kind of figure out what the picture actually means. So sometimes, I think the more words you replace with pictures, you'll be overcomplicating things. So only use pictures where it's appropriate I think is what I would say."* Consequently, developers must take into the consideration the cognitive abilities of target stakeholders and balance the number of pictures embedded within a single page to suit their needs.

Simplistic Interface

Digital Exclusion

Traditional barriers associated with the cost of AAC technologies [28] have been alleviated due to the emergence of mobile phone and tablet applications [28-30]. Nevertheless, adults with LDs have an increased chance of being affected by poverty [31] and may also be unable to afford (and maintain) cheaper mobile technologies. Participant 2 discussed the need to be aware of this problem when developing accessible applications, *"a lot of people with learning disabilities experience poverty and probably don't have iPhones and iPads and the touch screen stuff…I suppose the consideration for you is the impact digital exclusion [has] on this population."*

With little prior experience in using mobile technologies, many stakeholders may be unaware of the common actions required to operate touch screen technologies, such as swiping and pinching. The experts instead advocated for the implementation of more "intuitive" operations which emulate everyday movements e.g. tapping clickable elements. Strategies to supply mobile technologies to those in need will also have to be explored and participant 6 believes such funding may be accessible throughout Scotland, *"we have things*

called locally enhanced services or LES and if that included funding for tablet [technologies] with familiarity in training in how to use the tablet that could be worthwhile".

Declining Access to Support

Literature suggests that GPs often rely on caregivers to facilitate consultations involving patients with LDs [11], with a lack of appropriate training being cited for this dependency. Both practitioners interviewed agreed with this statement and believe that the knowledge held by carers plays a key role in forming a diagnosis, as discussed by participant six, *"if a person has got severe difficulties with communication, and you are not able to understand what the symptoms are, or how long they've been there you sometimes do have to rely on their overall behavior which you can ask the carers."*

This may ease the potential time restrictions placed on consultations, since caregivers are likely to use strategies that effectively extract information from the patient. However, participant five revealed that due to a lack of funding adults with MLDs are progressively having to make do without access to support, *"the hardest population would be the ones that don't have support. For adults with learning disabilities, the eligibility criteria tightened in most parts of the country [Scotland] and in some places it's like life and limb. Unless you've got some kind of critical risk to your health, you don't get support…So I can see why it would make sense to try to facilitate communication between doctor and patient."*

As a result, medical aids should aim to support the autonomous completion of tasks. Where this is not possible, alternative forms of support must be explored as you *may* not assume that caregivers will be present throughout the consultation.

Individualization

Portability

The technologies that are compatible with the software should also refrain from being restricted, as described by participant eight, *"if it's a tablet for example are you going to ask them to hold it? Is it going to be mounted or whatever… I would do it around the basis of the individual. You know, what [tablet] works [best] for that particular individual."* Instead, patients should be able to use the devices most suited to their complex needs e.g. those who have visual impairments may require a tablet with screen space that can display larger text. Medical practices may also be able to purchase the devices they deem to be most appropriate, thus increasing the likelihood of them investing in the application.

Co-Design

Much of the literature highlights the need to the include target stakeholders throughout the lifecycle of a system [34]. People with learning disabilities require a multitude of complex needs to be met - many of which may be unknown during the early design stages. Consequently, it is important to extract the views of stakeholders at multiple points throughout the project to ensure the developed artifacts appropriately cater for their needs. Four of the experts explicitly agreed with this and advocated for the development and evaluation of multiple technology probes in conjunction with a variety of stakeholders, as discussed by participant 2, *"obviously the more diverse that group [design group] is the better because you can the start to say well that works with that group of people but maybe*

not for that group of people." This conforms to the Complex Intervention Framework in which evidence is collected at multiple points throughout the lifecycle of an intervention and subsequently used to update the design of the aid.

Content Presentation

Increased Object Size

Around a third of people with learning disabilities have a significant visual impairment [48] that may affect their ability to interact with traditional user interfaces. As such, the experts advocated for the employment of general accessibility guidelines (such as the Web Content Accessibility Guidelines), meaning a minimum font size of 14 should be used. Nevertheless, the experts also recommended that text should be made as large as possible to occupy any screen space that may be available. Clickable objects should also be increased in size to accommodate for any visual or motor impairments that may be present.

Appropriate use of Color

Participant 10 revealed that in certain situations the use of color had an adverse effect on an individual's ability to complete a task, *"I tried using emoticons for someone on one occasion and they only saw the color. So they weren't able to recognize the facial expressions."* It is therefore important to conduct extensive user testing when color is implemented in novel ways to ensure its meaning is understood as intended.

Uniform User Interface

The need for consistency across the application was emphasized by participant 9, *"So maybe keep [the user's] options kind of limited and build it out in a kind of structure so that when you get to the end point you might have to go the long route rather than the shortcut."* As such, the pages embedded within clinical AAC applications should conform to a standard layout to ensure stakeholders are able to navigate across the aid effectively. An unstructured user interface will result in stakeholders having to learn multiple styles and functionalities between pages, thus increasing the overall complexity of the application. This may contribute to the high percentage of device abandonment mentioned previously [32].

Mobile Devices

The GPs revealed that they were willing to embed mobile devices within consultations providing the benefits of doing so are made explicit, as discussed by expert 6, *"I guess it's not the sort of thing which as yet has become routine practice. I don't know if anyone's ever come to me in a consultation with symptoms from an app but in theory if it was easy for the individual to use and they were going to use it I can't see any reason why that wouldn't be beneficial."*

Nevertheless, participant 6 believes that such aids may be met with resistance, *"I guess you're getting into the territory of using computer algorithms on symptoms to more or less tell you what's going on based on computer probabilities. That might be useful but I think it might be met with some resistance...ultimately people exist in a social context and many of their symptoms are socially patterned and an understanding of that context is crucial to interpreting those symptoms and you might not get that from a computer algorithm."*

Therefore, it is crucial to disseminate the evidence collected at various points throughout the Complex Intervention Framework to ensure the aids benefits are known and are accepted.

Results: Technology Probe Evaluation

This section covers those comments proposed by the experts involved in the usability study, which deemed to be less significant than those included in the main manuscript.

Communication Modalities

Highlighting the Skip Button's Purpose

A further option involving the use of audio was also proposed by two experts, and was summed up aptly by participant four, *"if most people ended up doing it by audio you could have a voice saying "or press the arrow for more options""*. Currently, the automatic audio function highlights and plays back all text displayed on completion of page loads. The improvement proposed would ensure that the skip button is also highlighted before a description of its function is played back. Once again, this matches the views of Medhi et al. who state "voice feedback should be provided for all functional units" [38]. Nevertheless, such a feature would only be appropriate for those patients who rely on audio and will not influence those who do not utilize the automatic playback function. Further options must be explored in regards to the image used to convey the skip button's functionality.

Individualization

Expert eight had an issue with the sensitivity of the screen and felt that this may cause serious complications for those stakeholders who have motor impairments, *"there was one [point] when it seemed to be very sensitive 'cause I didn't realize I had clicked on it. So, the problem with that is they might click the wrong one by mistake."* iOS and Android operating systems enable adjustments to be made to settings such as screen sensitivity and averaged activation [40]. However, the process of changing these settings may be too complex for people with learning disabilities, and an accessible solution to this must be explored.

Additional Features

Scale of Pain

Expert eight believed that some patients could confuse discomfort with pain in certain conditions such as tinnitus. One potential method to overcome this issue may be to introduce a Wong Baker smiley face pain scale during instances where the patient is required to disclose if they are in pain. Answers that gravitate towards the lower end of the scale may then be accepted as discomfort, at which point appropriate questions should be presented to the patient. This also conforms to the type of questions described expert 7, with GPs interested in the duration and intensity of symptoms.
